# Supplementary material for: LG5, a Novel Allele of EUI1, Regulates Grain Size and Flag Leaf Angle in Rice
Source: Plants (Basel). 2023 Feb 3;12(3):675. doi: 10.3390/plants12030675 (PMC9921835; doi:10.3390/plants12030675)
Supplement: Supplementary file 1 [file plants-12-00675-s001.zip › plants-2110093-supplementary.pdf]

Table S1. Sequences of primers used in this study

Name	Primer sequence (5' to 3')	Usage
	TTGCTTTGGAAACCCACACTCC	**Genetic analysis**
RM18985	GGATATCATCATCTCGTCGTCACC	
	GAGGTGGCGTTCTTGATGAGC	
RM18893	CATGCAGCTTTCACTTCAACTGG	
	CAAATTGCTAGAGCCTACCTTTCC	
RM18895	CGACTGTCTTTCAATACCGATGC	
	CTCCCAAAGGTCCTGGGAGAGC	
RM18900	CCCAAATCACACATACAGCCTCTCG	
	GGTGTCGCTTGTCAATTGTGTACC	
RM18882	AGCTATCAGGAGACGACCCAAGG	
	GAGAGGCATTTCCTCGAACACC	
RM18883	ATTGCAATCTCCCTTCACAACG	
	CATCATGTGGTAAGTGTGCAACG	
RM18639	GGTTGCGATGAGATTACGAGACC	
	CAGCCTTGGTAGCTGGATAATCG	
RM18717	CGTCTACTGCTCGAGAGAACTGTGG	
	TTGGTGGTGTAGATTCGGAGTGG	
RM18803	TCCTCCTCCTCCAAGAAACAATCC	
	CGGCTAAATCGTCATGTGTATGG	
RM18821	TCTCCCATCTTACATGTCCTCACC	
	GCTGGCTAAGCTAAGGCTATACCG	
RM18847	ATCTAGCTAGCAAGGAGGCAAGG	
	CCATGAATGATCGGAGATGAGAGC	
RM18903	CTCGTGCCACAGGTCCTTCC	
	CGCTCCGAAGTCTTCCTTCC	
RM18958	GTACTCCCTCATCCTCGTTTCG	
	TCGTTGCACTGAAGCAGACATGC	
RM19013	GTCCTTGCCGCCAAAGAATGG	
	CCTGATGATCCATGAAGTACAGACC	
RM19130	GAGGATTGCATTGACCGCTACC	
GW2	CAGCAGCGCATTCCCAGTTTTC	**qRT-PCR**
	GTGGTCAGCCGAGCACTCTC	
GL3.1	GCTCAAGGTCACCTGATCACTC	
	GAACGACCACAAGATCTCTGC	
GL7/GW7	CCCCTAGCATCGACACCAAG	
	CGGGTTCCAGCACTCCTCT	
TGW6	GGATTGATGCGAGTTGGT	
	GGATTGAAGAACGGTGACT	
GS2/GL2	TGCGTCCCTTCTTTGATGAGT	
	ACAGTTGGGTGCCTGAGAATG	
GS5	AGTGGACTGCTTCCAGGGAAG	

	CACGCAGTACCGAGAACTGA	
CYCA1;1	GTTTCGGTTGACGAGACGATGT	
	CGCTGCAAGGAACCTAGAACTG	
CYCA3;2	AGGTTGTCAAGATGGAGAGCGA	
	CGCTTTTTGTCTTCCTGGCA	
CYCB1;1	GGATTTACCGAGTCACAGCT	
	CAAGCGTGTCAAGATTCCAA	
CYCB2;2	CTCAAGGCTGCACAATCTGACA	
	GCATTGACGGCTGGAATTTG	
CDKA2	CGAGATTTGAAGCCCCAGAA	
	TCCGCGAGCTTCAATGAGTT	
CDKB2;1	AAGTTTGGCCAGGAGTGAGCA	
	TCAAGAGCATCAGCGTCGAGA	
LG5-Com1	ttcgagctcggtaccAGCTACCCACAGCCAGCTATATG	**Complementary vector construction**
	cgaagctgggccggcCGTCAGGCCACCGATTGATCT	
LG5-Com2	atcggtggcctgacgGCCGGCCCAGCTTCGATT	
	ctaattaacccatgaTTATCCTATGTGATGCTACATT	
LG5-Com3	catcacataggataaTCATGGGTTAATTAGGCTC	
	caggtcgactctagaACTGTCTTCAGCACACGTA	
LG5-OE	tcgcgagctcggtaccATGGAGAGCTTCTTCGTCTTCT	**Over-expression construction**
	gcctgcaggtcgactctagaTTAATTATGAGAACTATGCACG	
LG5-RNAi1	gacctcgagggtaccATGGAGAGCTTCTTCGTC	**RNA interference**
	gactctagaggatccTTAATTATGAGAACTATGCACG	
LG5-RNAi2	cagatcgatactagtATGGAGAGCTTCTTCGTC	
	gggaaattcgagctcTTAATTATGAGAACTATGCACG	
LG5-GFP	gaggggggggcccggtaccATGGAGAGCTTCTTCGTCTTC	**Subcellular localization**
	cccttgctcaccatggtaccATTATGAGAACTATGCACGGGT	

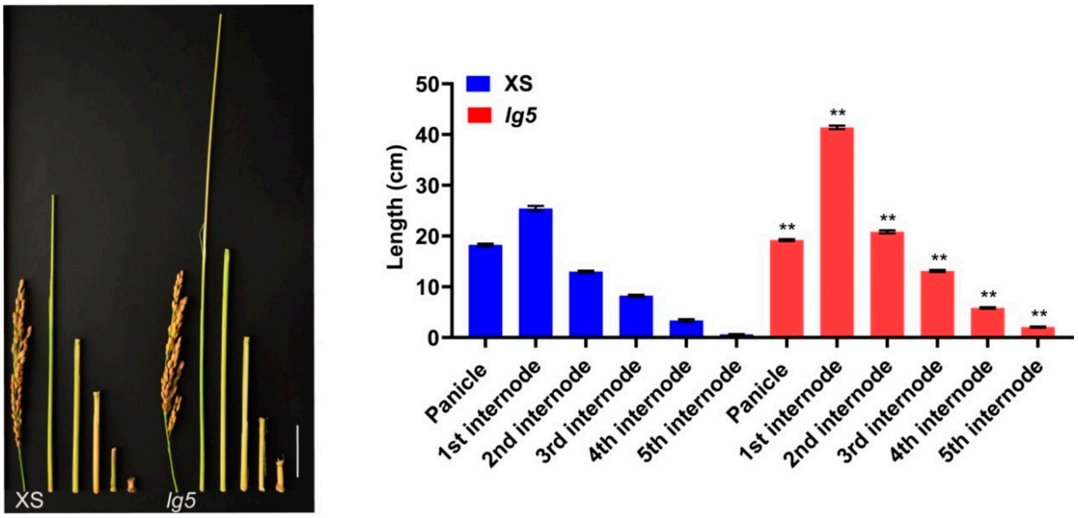

Figure S1. Length of panicle and internode, bar=5cm. Data are given as means ± SD. * and ** indicate $P <$ 0.05 and $P < 0.01$ by Student's *t*-test.

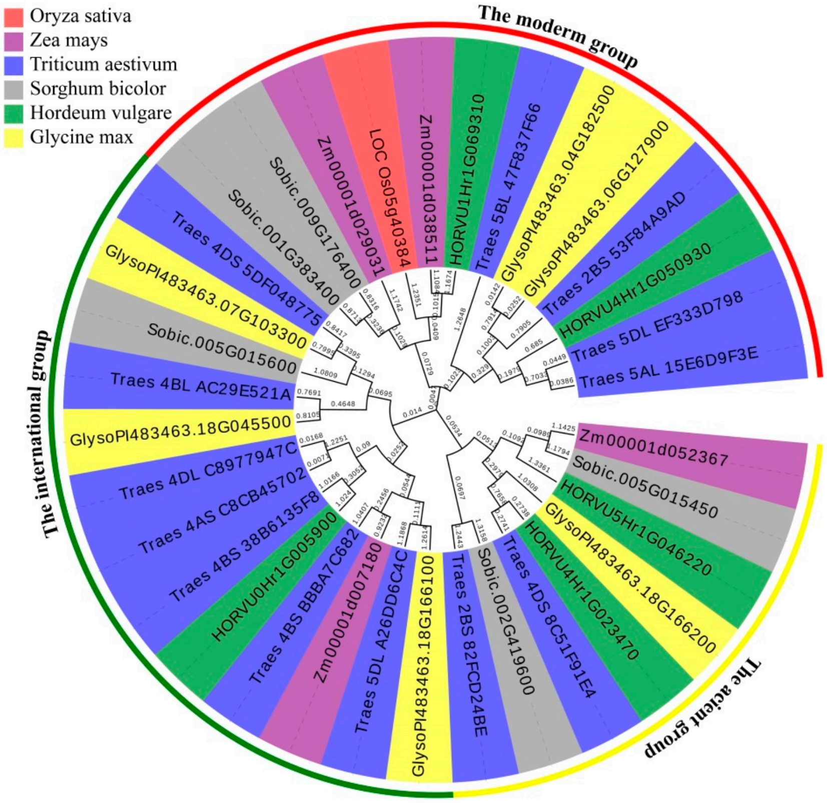

Figure S2. Evolutionary tree of *LG5* in different cereal crops, the tree was constructed by the neighbor-joining method. 33 species from *Oryza sativa* (*Os*), *Zea mays* (*Zm*), *Triticum aestivum* (*Traes*), *Sorghum bicolor* (*Sobic*), *Hordeum vulgare* (*Horv*u), *Glycine max* (*Glysopl*) were constructed by Mega 6 and divided into 3 groups.
